# A Checklist of the Herpetofauna of Nusa Kambangan Island, Central Java, Indonesia

**DOI:** 10.21315/tlsr2022.33.2.6

**Published:** 2022-07-15

**Authors:** Nia Kurniawan, Luhur Septiadi, Muhammad Fathoni, Ahmad Muammar Kadafi, Agung Pramana Warih Marhendra

**Affiliations:** 1 Department of Biology, Faculty of Mathematics and Natural Sciences, Universitas Brawijaya, Malang 65145, East Java, Indonesia; 2 Department of Biology, Faculty of Science, Chulalongkorn University, Pathumwan 10330, Bangkok, Thailand; 3 Department of Biology, Faculty of Mathematics and Natural Sciences, Universitas Palangka Raya, Palangka Raya 74874, Central Kalimantan, Indonesia

**Keywords:** Amphibians, Lowland Forest, New Record, Reptiles, Threatened Species

## Abstract

An inventory of herpetofauna species from western part of Nusa Kambangan Island, Central Java, Indonesia, is presented. There are 43 herpetofauna species reported (16 amphibians and 27 reptiles). This study confirmed new distribution record and list some of threatened species. In light of the imminent human disturbances on Nusa Kambangan Island, a conservation plan is urgently needed.

**Highlights**

- The first comprehensive survey of Nusa Kambangan Island’s herpetofauna from the west part.
- The confirmation and distribution record of a several herpetofauna species are reported.
- Lowland forest type 1 became the most significant habitat due to its high species richness of herpetofauna, but it was threatened by human disturbance.

## INTRODUCTION

Indonesia, as a biodiversity hotspot and the largest archipelagic country in Southeast Asia, is home to a wide range of tropic fauna, including herpetofauna (Roos *et al*. 2004). Herpetofauna discoveries began primarily in Java in the 19th century (Boulenger 1890; de Rooij 1917; van Kampen 1923; Kopstein 1930; de Haas 1941) and expedited our understanding of Indonesia’s species diversity. Recent decades have seen a rapid increase in the number of newly described herpetofauna species, particularly for amphibians, e.g., Megophrydae (Hamidy & Matsui 2010; 2014; Hamidy *et al*. 2012; Eto *et al*. 2018; Munir *et al*. 2018; 2019; 2021b), Rhacophoridae (Matsui *et al*. 2014; Hamidy & Kurniati 2015; Wostl *et al*. 2017b; Mediyansyah *et al*. 2019; Munir *et al*. 2021a), Microhylidae (Matsui *et al*. 2013; Atmaja *et al*. 2019; Munir *et al*. 2020; Eprilurahman *et al*. 2021), Bufonidae (Smart *et al*. 2017; Hamidy *et al*. 2018; Sarker *et al*. 2019), Ranidae (Matsui & Hamidy 2012; Arifin *et al*. 2018), Dicroglossidae (Iskandar *et al*. 2011a; Mcleod *et al*. 2011); and reptiles, e.g., Gekkonidae including *Cyrtodactylus* (Iskandar *et al*. 2011b; Riyanto *et al*. 2018a; 2018b; 2021), *Cnemaspis* (Amarasinghe *et al*. 2015a; Riyanto *et al*. 2017; Iskandar *et al*. 2017), *Hemiphyllodactylus* (Grismer *et al*. 2014), and *Lepidodactylus* (Stubbs *et al*. 2017); Agamidae (Harvey *et al*. 2014; 2017a; 2017b; 2018; 2019), Colubridae (Vogel *et al*. 2014; Amarasinghe *et al*. 2015b; 2021; Wostl *et al*. 2017a), and Cylindrophiidae (Kieckbusch *et al*. 2016); which highlight the fact that many enigmatic, elusive, and cryptic species are waiting to be discovered. Although the region of Sumatra, Borneo, Celebes, Moluccas and Java has been extensively surveyed (David & Vogel 1996; Inger *et al*. 2005; de Lang & Vogel 2006; Kurniawan *et al*. 2021; Kusrini *et al*. 2021), the primary focus has been on areas with relatively high diversity. Due to gaps in survey efforts, many areas of Java’s herpetofauna remain unexplored (Kurniawan *et al*. 2021; Kusrini *et al*. 2021).

Nusa Kambangan Island is located in Southern Central Java and has a diverse range of ecological landscapes (Abdiyani 2008; Sulton *et al*. 2018) but has received little attention due to a lack of intensive surveys. Prior surveys in Nusa Kambangan Island focused on the diversity of avian (Suripto & Hamidy 2006), lepidopteran (Peggie 2014; Sutrisno 2007), and dipterocarp (Dwiyanti *et al*. 2014) species, with only a few herpetofauna species reported (Iskandar 1998; IUCN SSC Amphibian Specialist Group 2018). The diversity of herpetofauna on Nusa Kambangan Island has received less attention than in surveys conducted in Central Java (see Iskandar 1998; Riyanto *et al*. 2009; Toni *et al*. 2010; Eprilurahman *et al*. 2010; Riyanto 2010; Qurniawan & Eprilurahman 2012; Riyanto & Trilaksono 2012; Mumpuni 2014; Subeno 2018). Thus, baseline data on herpetofauna species are critical for future management of Nusa Kambangan Island’s conservation.

Herpetofauna baseline data can be used as a preliminary step for investigating environmental changes and habitat degradation. In this study, we report on the diversity of herpetofauna found on the west part of Nusa Kambangan Island. These data will provide essential information about herpetofauna species and will aid in the future management of the conservation of Nusa Kambangan Island.

## MATERIALS AND METHODS

### Study Area

**Figure 1:**
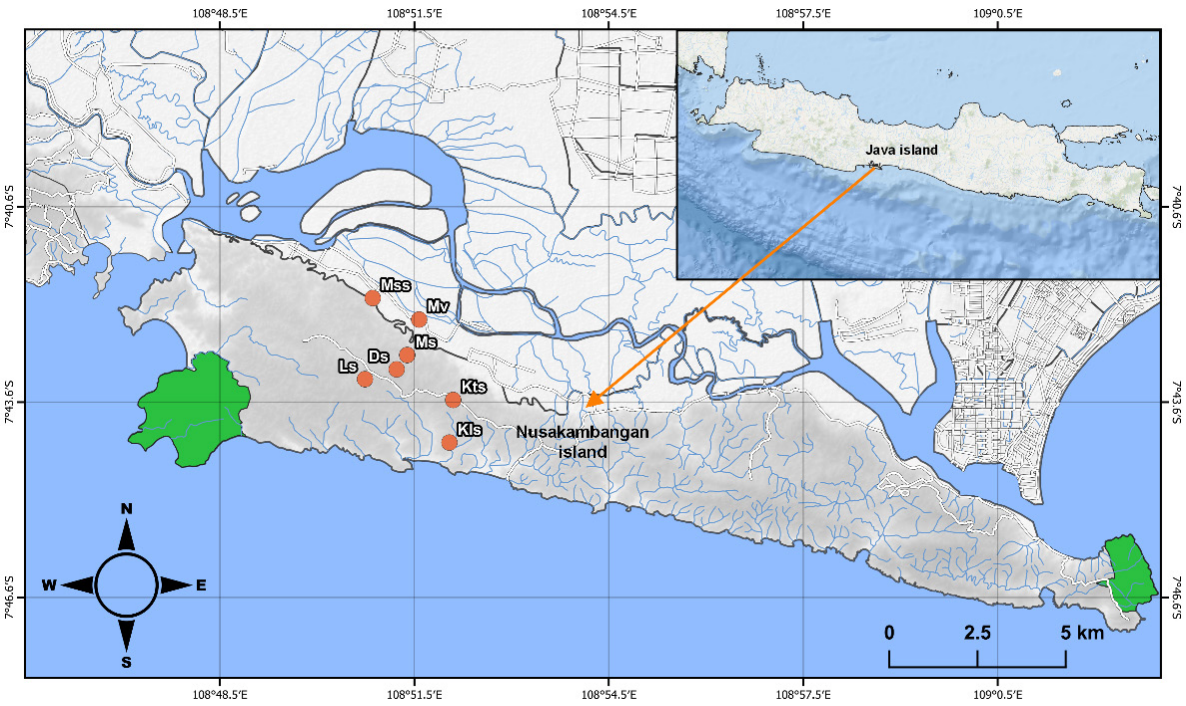
Map of survey sites in the western part of Nusa Kambangan Island showing surveyed stream (orange dot) including: (Mss) Masigit Sela stream, (Kts) Ketapang stream, (Kls) Kalidua stream, (Ds) Darmoko stream, (Ms) Mangunjaya stream, (Ls) Lapas stream and (Mv) Motean village. The areas in the west and east of island (green areas) show the Nusa Kambangan Nature Preserve.

**Figure 2:**
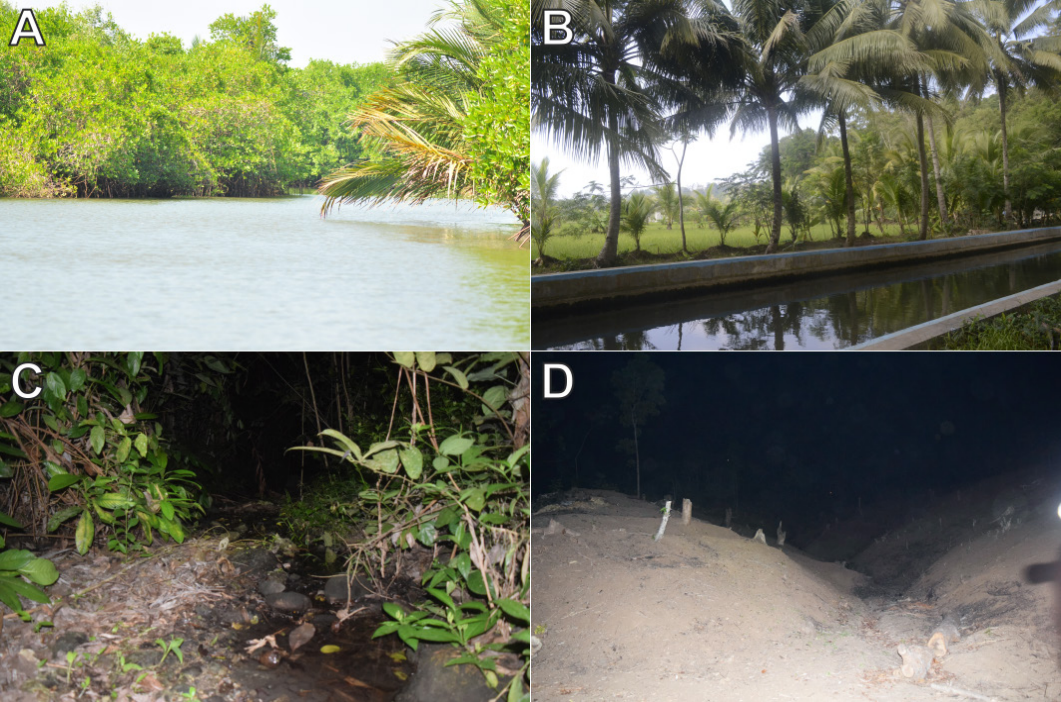
Examples of herpetofauna habitats in the western part of Nusa Kambangan Island including (A) estuaries or mangrove forest, (B) drainage canals, (C) lowland forests and flowing freshwater, and (D) harvested timber forest production.

Nusa Kambangan Island encompasses an area of 210 km^2^ (KKP–Direktori Pulau-Pulau Kecil Indonesia 2021) and is considered an ecologically significant area due to its diverse ecological landscape. This island was located in the southern region of Central Java, whereas the island’s natural reserve areas are located in the island’s eastern and western regions (Fig. 1). It is characterised by lowland habitats such as coastal and mangrove forests (Fig. 2A) and forest with karst caves and flowing freshwater (Fig. 2C), as well as an elevation range of up to 150 meters above sea level (m asl). The island faces human disturbance and environmental degradation as a result of logging (Fig. 2D), land conversion, mining, and wildlife trade (Whitten *et al*. 1999; Dharmawan *et al*. 2017; Ayuningrum & Purnaweni 2018).

Our survey was conducted on Nusa Kambangan Island’s west part, specifically in the Kampung Laut District, Cilacap Regency. To investigate the region’s herpetofauna diversity, survey sites included areas with natural habitat, even those with a high degree of human disturbance (e.g., logging areas, agricultural areas, and mining areas). We surveyed several sites including (datum: WGS 84): Masigit Sela stream (Mss; 7°41′57.70″S, 108°50′53.90″E), Motean village (Mv; 7°42′17.49″S, 108°51′36.88″E), Mangunjaya stream (Ms; 7°42′50.17″S, 108°51′25.86″E), Lapas stream (Ls; 7°43′12.40″S, 108°50′46.80″E), Darmoko stream (Ds; 7°43′3.34″S, 108°51′16.08″E), Ketapang stream (Kts; 7°43′31.60″S; 108°52′8.40″E), and Kalidua stream (Kls; 7°44′10.80″S, 108°52′4.90″E) (Fig. 1).

### Field Sampling

We conducted a standard Visual Encounter Survey using purposive sampling methods (Kusrini 2008; Dorcas & Wilson 2009), by night (1700 pm–2400 am), from 7–8 December 2018, 3–4 March 2019, and 27–29 June 2020. Three workers surveyed each stream in order to increase our effectiveness in finding herpetofauna species (Kusrini 2008). Each surveyor was equipped with a headlamp and other required safety equipment. We collected data on the individual, elevation, location, habitat, natural history, as well as documentation of the species (*in-situ*).

### Specimens Identification and Collection

We identified the herpetofauna species by examining external morphological characteristics and consulting previously published literature (Boulenger 1890; de Rooij 1917; van Kampen 1923; de Haas 1941; Inger 1966; Iskandar 1998; Iskandar & Colijn 2000; Das 2010; de Lang 2017; Frost 2020; Uetz *et al*. 2016). The collected voucher specimens were euthanised with 7.5% benzocaine, fixed in 10% formalin for 24 h, preserved in 70% ethanol, and accessioned at the Laboratory of Animal Diversity and Ecology Collection, Biology Department, Universitas Brawijaya, Indonesia (NK). For further molecular analysis, a tissue sample was collected in 95% ethanol.

### Data Analysis

We classified herpetofauna species by family and assessed their conservation status using the Indonesian Ministry of Environment and Forestry’s list of national protected fauna and flora (PERMEN LHK; Ministry of Environment and Forestry 2018). Additionally, we consulted the IUCN Red List of Threatened Species (IUCN 2020) to determine the species’ conservation status. Furthermore, we consulted the Convention on International Trade in Endangered Species of Wild Fauna and Flora (CITES 2021) for the regulation of international trade of species under threat. Following their respective categorisation, the data were analysed for community structure using the diversity index (H’; Shannon-Wiener) (Hill 1973), abundance, taxa richness, and dominance index (D; Simpson index) (Hill *et al*. 2005). We used Zar’s modified *t*-tests, also known as Hutcheson *t*-tests, to determine the significance difference of species diversity between sites (Hutcheson 1970; Das *et al*. 2007). MS-Excel for macOS was used to perform all analyses.

We plot the species richness across vegetational types and/or habitat complexes that were encountered during the study by referring to the ecology of the islands (Whitten *et al*. 1999). Vegetational types and/or habitat complexes were delimited based on floristic and structural differences along the elevational gradient. Vegetational types and/or habitat complexes were summarised into following categories:

- *Mangrove forest type 1*, is a forest composed primarily of mangroves (e.g., *Avicennia* sp., *Rhizophora* sp., *Sonneratia* sp.) that inhabits mudflats (coastal wetlands that form in intertidal areas where tides or rivers deposit sediments). The disturbance caused by humans to this habitat complex is relatively low.
- *Mangrove forest type 2*, is a forest composed of mangroves (e.g., *Xilocarpus* sp.) which has been associated with other lowland forest vegetations, characterised by water canals and small streams. The disturbance caused by humans is more pronounced in this habitat complex than in mangrove forest type 1 (e.g., docking boat, waste load).
- *Fishpond and settlement area*, is a rural area characterised by the presence of small communities. The areas were adjacent to water canals and composed of shrubs, palm and coconut trees, fishponds, and human settlements. The disturbance caused by humans to this habitat complex is profound.
- *Paddy field and timber production forest*, is an area characterised by the presence of paddy fields and sparsely timber production forest that have not been logged by the communities. The area is not overly steep, and the disturbance caused by humans is significant.
- *Edges between agriculture and forest*, is the border between two distinct habitats, the agricultural area and the forest. The hills were excessively steep, the timber production forest was still present, and human disturbance is less significant than in paddy fields and timber production forests, as the area is distant from human settlement areas.
- *Tributary stream and degraded areas*, is an area primarily made up of small water streams that disembogued into canals; some areas are open due to harvested timber forest, and the water streams are mostly murky due to erosion and soil degradation. This habitat was typically found between the edges of agriculture and forest and lowland forest type 1; areas with a high level of human disturbance.
- *Lowland forest type 1*, is a natural habitat that is flowed by clear freshwater from karst caves, dense canopies, and is covered in a various vegetation. Typically, this area is accessible to locals in search of forest resources.
- *Lowland forest type 2*, is a pristine habitat fed by freshwater, with rocky terrain, dense canopies, and a wide and varied vegetation cover. The canopy was so dense that sunlight could not reach the forest floor thoroughly. This area was so secluded that the locals considered it inaccessible.

## RESULTS

### Species Diversity and Conservation Status

**Figure 3:**
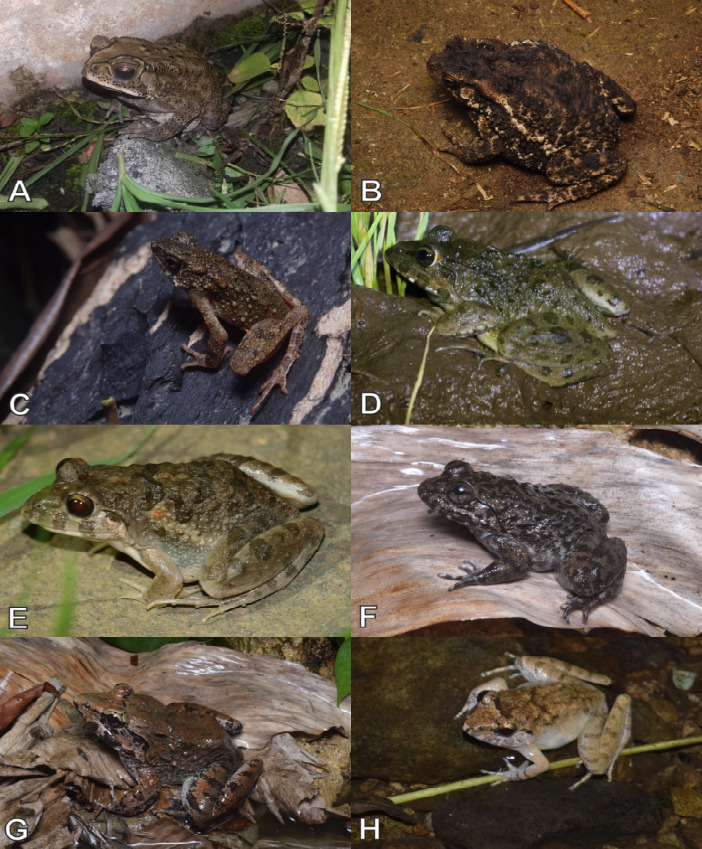
Anurans found on the western part of Nusa Kambangan Island including (A) *Duttaphrynus melanostictus*, (B) *Ingerophrynus biporcatus*, (C) *Leptophryne borbonica*, (D) *Fejervarya cancrivora*, (E) *Fejervarya limnocharis*, (F) *Limnonectes kuhlii*, (G) *Limnonectes macrodon*, and (H) *Limnonectes microdiscus*.

**Figure 4:**
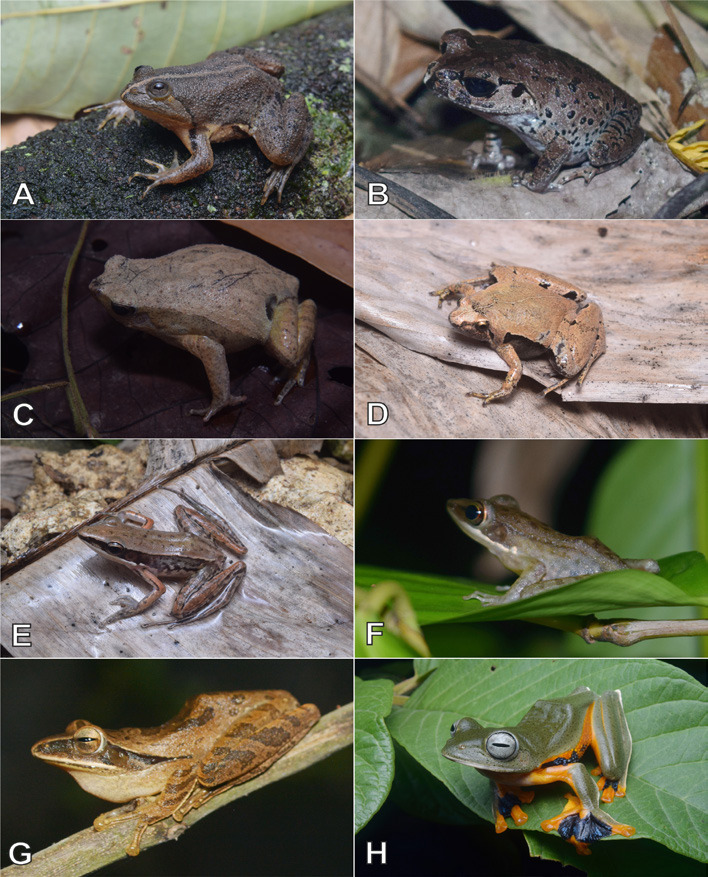
Anurans found on the western part of Nusa Kambangan Island including (A) *Occidozyga lima*, (B) *Leptobrachium hasseltii*, (C) *Kalophrynus minusculus*, (D) *Microhyla achatina*, (E) *Indosylvirana nicobariensis*, (F) *Chalcorana chalconota*, (G) *Polypedates leucomystax*, and (H) *Rhacophorus reinwardtii*.

**Figure 5:**
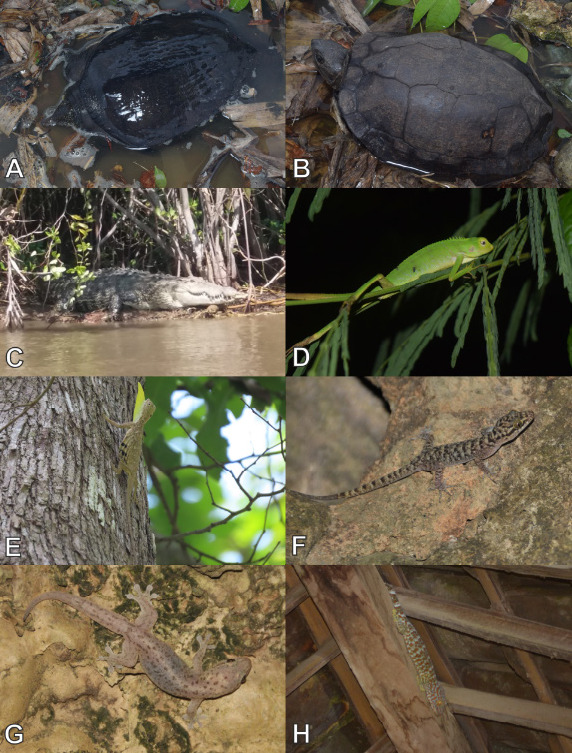
Turtles, crocodiles, and lizards found on the western part of Nusa Kambangan Island including (A) *Amyda cartilaginea*, (B) *Cyclemys dentata*, (C) *Crocodylus porosus*, (D) *Bronchocela jubata*, (E) *Draco volans*, (F) *Cyrtodactylus marmoratus*, (G) *Gehyra mutilata*, and (H) *Gekko gecko*.

**Figure 6:**
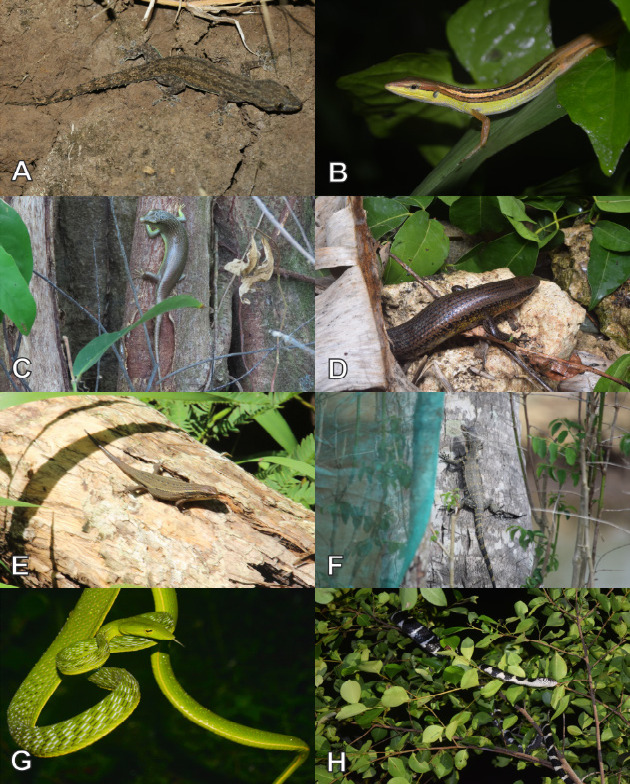
Lizards and snakes found on the western part of Nusa Kambangan Island including (A) *Hemidactylus frenatus*, (B) *Takydromus sexlineatus*, (C) *Dasia olivacea*, (D) *Eutropis multifasciata*, (E) *Eutropis rugifera*, (F) *Varanus salvator*, (G) *Ahaetulla prasina*, and (H) *Boiga dendrophila*.

**Figure 7:**
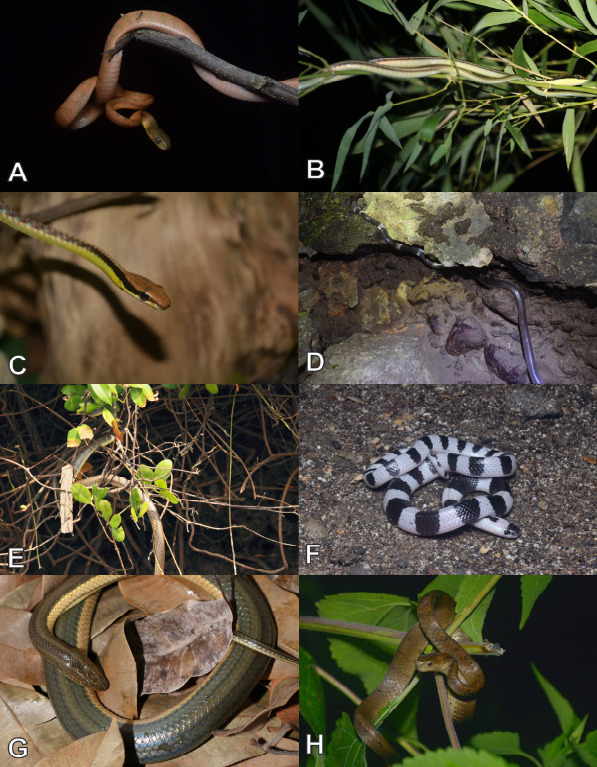
Snakes found on the western part of Nusa Kambangan Island including (A) *Boiga nigriceps*, (B) *Dendrelaphis pictus*, (C) *Dendrelaphis underwoodi*, (D) *Lycodon subcinctus*, (E) *Ptyas korros*, (F) *Bungarus candidus*, (G) *Enhydris enhydris*, and (H) *Pareas carinatus*.

**Figure 8:**
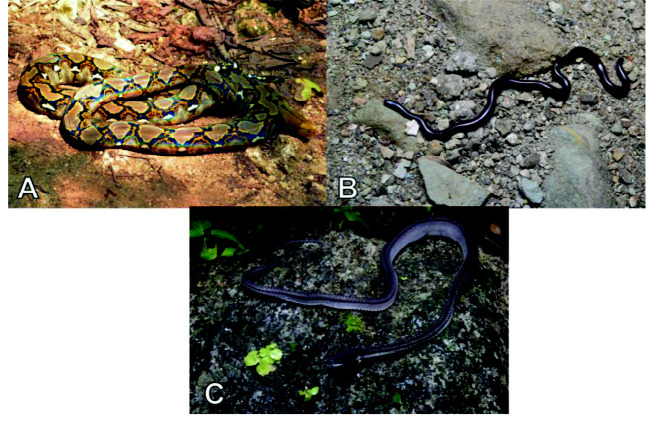
Snakes found on the western part of Nusa Kambangan Island including (A) *Malayopython reticulatus*, (B) *Indotyphlops braminus*, and (C) *Xenodermus javanicus*.

Currently, a total of 43 herpetofauna consisting of 16 amphibians and 27 reptiles (Figs. 3–8) are recorded from the western part of Nusa Kambangan Island. Three threatened species were discovered, according to the IUCN conservation status (2020), including *Rhacophorus reinwardtii* (Near Threatened; NT), *Cyclemys dentata* (NT), and *Amyda cartilaginea* (Vulnerable). Six highly traded species categorised in Appendix II were identified using CITES (2021), including the *Gekko gecko*, *Malayopython reticulatus*, *C. dentata*, *A. cartilaginea*, *Varanus salvator*, and *Crocodylus porosus*. According to the PERMEN LHK list, our surveys identified one protected species, *C. porosus*. Table 1 contains an annotated checklist of herpetofauna species, as well as a species inventory as follows.

## SPECIES INVENTORY

### Amphibia

#### Family Bufonidae

*Duttaphrynus melanostictus* (Schneider 1799) (Fig. 3A)

**Common name:** Asian Common Toad, Asian Black-spined Toad, Spectacled Toad and Southeast Asian Toad

**Type locality:** India

**Distribution and habitat:** This toad is found throughout Indonesia (Sumatra, Java, and Borneo), Singapore, Malaysia, Brunei Darussalam, Thailand, Cambodia, Myanmar, Lao PDR, Vietnam, India (including Andaman and Nicobar Islands), Bangladesh, Pakistan, Sri Lanka, Nepal, Bhutan, Maldives, Hong Kong and China (including Taiwan and Hainan); it is also found in Bali, Madagascar, Sulawesi, Ambon and New Guinea. Their habitat is inextricably linked to human settlement and disturbed area (Othman *et al*. 2020; Frost 2020).

**Local remarks:** Numerous individuals were spotted along the walkway and in settlements within Mv.

*Ingerophrynus biporcatus* (Gravenhorst 1829) (Fig. 3B)

**Common name:** Indonesian Toad, Sunda Ridge-headed Toad and Crested Toad

**Type locality:** Indonesia (Java)

**Distribution and habitat:** This species is found throughout Indonesia, including Sumatra, Java, Bali and Nusa Tenggara. It may have been introduced to Sulawesi as well. It is found in primary and secondary forest at elevations of up to 700 m asl. The habitat is closely linked to human activity. However, in Java, it seems that this species is restricted to forested areas (Reilly *et al*. 2020; Frost 2020).

**Local remarks:** This toad was observed across the floor of the secondary forest, which is densely covered in leaf litter and is located near the Mss.

*Leptophryne borbonica* (Tschudi 1838) (Fig. 3C)

**Common name:** Bourbon Toad, Java Tree Toad, Slender-legged Toad and Hour-glass Toad

**Type locality:** Indonesia (West Java)

**Distribution and habitat:** It is known to occur on the Indonesian islands of Java, Kalimantan and Sumatra. There have also been reports of occurrences in Sarawak, Sabah, Peninsular Malaysia and Peninsular Thailand. It is mainly found in the leaf litter of moist forests less than 400 m asl. It is occasionally seen in marshy or wet areas with clear, slow-moving waters (Erfanda *et al*. 2019; Poyarkov *et al*. 2021; Frost 2020).

**Local remarks:** Several individuals were observed only at the Kls, along a shallow stream and along the forest cliffs of a primary forest.

#### Family Dicroglossidae

*Fejervarya cancrivora* (Gravenhorst 1829) (Fig. 3D)

**Common name:** Java Wart Frog, Javan Wart Frog, Mangrove Frog, Marsh Frog, Field Frog, Rice Field Frog, Crab-eating Frog, Brackish Water Frog, Southern Grassfrog, Crab-eating Frog, Gulf Coast Frog and Rajah frog

**Type locality:** Indonesia (Java)

**Distribution and habitat:** It distributed throughout Thailand (including the provinces of Bangkok and Cholburi), Malaysia and Indonesia (including Sumatra, West Java, Central Java and Bali) and was introduced into New Guinea and Guam. This species is found primarily in lowland rainforests, forest edges, lower montane forests, monsoon forests, brackish waterways, mangrove swamps and agricultural areas on Bali (Mckay 2006; Frost 2020).

**Local remarks:** The frogs were found in muddy waters beneath gaps between mangrove roots and dense shrubs near brackish water, not far from Mv and Mss.

*Fejervarya limnocharis* (Gravenhorst 1829) (Fig. 3E)

**Common name:** Paddy Field Frog, Cricket Frog, Grass Frog, Field Frog, Paddy Frog, Rice Frog, Ricefield Frog, Terrestrial Frog, White-lined Frog, Indian Cricket Frog and Boie’s Wart Frog

**Type locality:** Indonesia (Java)

**Distribution and habitat:** It is found throughout South and East Asia, as well as Southeast Asia. Sumatra, Java, Kalimantan and Sulawesi comprise the Indonesian distribution. They inhabit wetlands, forests, savannah, grassland and man-made habitats such as paddy fields and urban areas (Frost 2020).

**Local remarks:** Numerous individuals were spotted on drainage canals in the vicinity of human settlements near Mss and Mv. This species is also found in Kts and Ds on the bank of a small stream.

*Limnonectes kuhlii* (Tschudi 1838) (Fig. 3F)

**Common name:** Kuhl’s Fanged Frog, Kuhl’s Creek Frog, Kuhl’s Wart Frog, Kuhl’s Frog, Large-headed Frog, Big-headed Frog and Big-headed Mountain Frog

**Type locality:** Indonesia (Java)

**Distribution and habitat:** This species is endemic to Java’s mountainous forest, where it is found primarily near slow- or moderate-moving water. They are frequently found near shallow water banks and crab nesting holes (Frost 2020).

**Local remarks:** In Kts and Ms, this species was submerged in a rocky stream under the dense canopy.

*Limnonectes macrodon* (Duméril & Bibron 1841) (Fig. 3G)

**Common name:** Stone Creek Frog, Giant Javan Frog, Brown Mountain Frog, Malaya Wart Frog and Malayan Giant Bullfrog

**Type locality:** Indonesia (Java and *“Celebes”* = Sulawesi)

**Distribution and habitat:** This species is known to occur in Java and Sumatra of Indonesia. It is common along rivers and clear streams (Iskandar 1998; Frost 2020).

**Local remarks:** They were observed at the rocky stream, concealing themselves beneath the leaf litter that covers the stream’s side in Kts and Ms.

*Limnonectes microdiscus* (Boettger 1892) (Fig. 3H)

**Common name:** Indonesia Wart Frog and Pygmy Creek Frog

**Type locality:** Indonesia (Tengger, East Java)

**Distribution and habitat:** This species is endemic to the Indonesian island of Java. It is found only in forested areas between 0–1400 m asl (Iskandar 1998; Frost 2020).

**Local remarks:** This species was discovered near the Ds and Ms in a rocky stream.

*Occidozyga lima* (Gravenhorst 1829) (Fig. 4A)

**Common name:** Puddle Frog, Green Puddle Frog, Common Puddle Frog, Gray-green Puddlefrog, Green Floating Frog, Aquatic Frog, Pointed-tongue Floating Frog and Rough-skinned Floating Frog

**Type locality:** Indonesia (Java)

**Distribution and habitat:** This species is found throughout India, Bangladesh, Myanmar, Thailand, Cambodia and Lao PDR. Additionally, it is found throughout southern China, Vietnam, Malaysia and Indonesia (i.e., Java and Bali). They are abundant in rice fields, submerged and floating with their bulging eyes visible above the surface of the water (Flury *et al*. 2021; Frost 2020).

**Local remarks:** This species was observed in Kts and Ls near a shallow stream and a sloping rock.

#### Family Megophrydae

*Leptobrachium hasseltii* (Tschudi 1838) (Fig. 4B)

**Common name:** Hasselt’s Litter Frog, Tschudi’s Frog, Java Spadefoot Toad and Red-legged Crawl Frog

**Type locality:** Indonesia (Java)

**Distribution and habitat:** This species is found only on the Indonesian islands of Java, Madura, Bali and Kangean. It is primarily found in forested areas and is easily found at elevations greater than sea level (Iskandar 1998; Frost 2020).

**Local remarks:** It was primarily discovered on a damp rock, but was also found scattered throughout the walkway and shallow stream in Ms and Ls.

#### Family Microhylidae

*Kalophrynus minusculus* (Iskandar 1998) (Fig. 4C)

**Common name:** Small Sticky Frog and Dwarf Sticky Frog

**Type locality:** Indonesia (Ujung Kulon, West Java)

**Distribution and habitat:** This species is found only in West Java and western Sumatra, Indonesia. They are found in forest habitats at lower elevations (Iskandar 1998; Frost 2020).

**Local remarks:** This species was discovered in the primary forest near Kls and Ls at sea-level altitudes, above fallen dried palm trees. This observation corroborated the previous record of distribution (IUCN SSC Amphibian Specialist Group 2018).

*Microhyla achatina* (Tschudi 1838) (Fig. 4D)

**Common name:** Javanese Narrow-mouthed Frog, Javan Chorus Frog and Java Rice Frog

**Type locality:** Indonesia (Java)

**Distribution and habitat:** It is only found in Java of Indonesia. This species is restricted to primary and secondary forests, though it is occasionally encountered near human settlements up to an elevation of 1,600 m asl (Frost 2020).

**Local remarks:** In Mss and Ls, this species was common in leaf litter near water sources.

#### Family Ranidae

*Indosylvirana nicobariensis* (Stoliczka 1870) (Fig. 4E)

**Common name:** Cricket Frog, Nicobar Frog, Nicobarese Frog, Nicobar Cricket Frog and Nicobar Island Frog

**Type locality:** Indonesia (Java)

**Distribution and habitat:** It is found in Philippines and Indonesia (specifically, Sumatra, Java and Kalimantan), as well as Brunei Darussalam, Thailand and Malaysia (including Nicobar Islands). They are typically found near disturbed areas and slow-moving waters at the forest edge. It is found in Java up to 1,500 m asl (Lalronunga *et al*. 2021; Frost 2020).

**Local remarks:** This species was discovered in the Ds and Ls above the sloping rocks.

*Chalcorana chalconota* (Schlegel 1837a) (Fig. 4F)

**Common name:** White-lipped Frog, Brown Stream Frog, Copper-cheeked Frog and Schlegel’s Frog

**Type locality:** Indonesia (Java)

**Distribution and habitat:** It is found in Java, Sumatra and Bali in Indonesia. It is found up to 1,200 m asl. It is frequently encountered in human settlements adjacent to bodies of water, where it thrives in stagnant waters, leaves, and nearby vegetation (Frost 2020).

**Remarks:** Except for Mv, this frog was easily encountered in all of the streams surveyed.

#### Family Rhacophoridae

*Polypedates leucomystax* (Gravenhorst 1829) (Fig. 4G)

**Common name:** Common Tree Frog, Java Whipping Frog, Four-lined Tree Frog, White-lipped Tree Frog, Brown Tree Frog, Stripe Tree Frog, Malayan Tree Frog, Bamboo Tree Frog, Malayan House Frog, House Tree Frog and Jar Tree Frog

**Type locality:** Indonesia (Java)

**Distribution and habitat:** Southern China, Indo-China, India, Philippines and Indonesia are all parts of this species’ range. This species is frequently found in marshes and secondary forest ecosystems. This species is tolerant of areas subject to high levels of human disturbance (Frost 2020).

**Local remarks:** This species was discovered near water sources on leaf and tree branches. Except for Mv, this frog was easily encountered in all of the streams surveyed.

*Rhacophorus reinwardtii* (Schlegel 1840) (Fig. 4H)

**Common name:** Reinwardt’s Tree Frog, Reinwardt’s Flying Frog, Reinwardt’s Gliding Frog, Reinwardti’s Frog, Black-webbed Tree Frog, Green Flying-frog and Small Flying Tree Frog

**Type locality:** Indonesia (Java)

**Distribution and habitat:** This species is found only on the island of Java, Indonesia. It is frequently encountered in primary and secondary forest habitats. It prefers higher elevations between 250 m asl–1,200 m asl (O’Connell *et al*. 2018; Frost 2020).

**Local remarks:** The tree frog was observed above the leaf, well camouflaged within the vegetation, at a height of approximately 1.5 m above the ground and covered in dense canopy toward Ls.

### Reptile

#### Family Tryonicidae

*Amyda cartilaginea* (Boddaert 1770) (Fig. 5A)

**Common name:** Asiatic Softshell Turtle and Black-rayed Soft-shelled Turtle

**Type locality:** N/A

**Distribution and habitat:** This softshell turtle is found in Indonesia (including Java, Sumatra, Kalimantan and Sulawesi), Malaysia, Singapore and India (including Assam), and was most likely introduced to Indonesia’s Lesser Sunda and Sulawesi, as well as Yunnan of China. They are found in a variety of freshwater habitats and peat swamps (Uetz *et al*. 2016).

**Local remarks:** Adults have been observed in muddy water puddles, sandy water puddles and on the surface. This species was discovered in the Ls.

#### Family Geoemydidae

*Cyclemys dentata* (Gray 1831a) (Fig. 5B)

**Common name:** Asian Leaf Turtle

**Type locality:** Indonesia (Java)

**Distribution and habitat:** Freshwater turtles are found throughout Indonesia (Sumatra, Java, Kalimantan and Bali), the southern Malay Peninsula, and Phillipines (including Palawan and Calamian islands). They prefer low plains and are frequently encountered in small rivers, shallow streams, and puddles (Fritz *et al*. 2008; Uetz *et al*. 2016).

**Local remarks:** The freshwater turtle was discovered in a muddy puddle with a depth of approximately 50 cm–70 cm and water that was slowly moving and covered in damp leaves. Because the canopy was too dense, the populations remained unscathed by human disturbances. This species was discovered in the Kts, Ds, Ms, and Ls.

#### Family Crocodilidae

*Crocodylus porosus* (Schneider 1801) (Fig. 5C)

**Common name:** Saltwater crocodile

**Type locality:** Indonesia (Java)

**Distribution and habitat:** This huge reptile is found throughout Australia, Palau, Papua New Guinea, Philippines, Vanuatu, Micronesia, Indonesia (specifically, Java, Sulawesi and Nusa Tenggara), Malaysia, Brunei Darussalam, Singapore, Vietnam, Myanmar, Thailand, Cambodia, India, Sri Lanka and Bangladesh. This species is commonly found in estuaries (Uetz *et al*. 2016).

**Local remarks:** According to locals, seven individuals were observed basking beneath the mangroves. From the estuaries in the northern part of Mss, we were only able to photograph one individual.

#### Family Agamidae

*Bronchocela jubata* (Duméril & Bibron 1837) (Fig. 5D)

**Common name:** Maned forest lizard

**Type locality:** Indonesia (Java)

**Distribution and habitat:** This species is found throughout Indonesia (Java, Bali, Sulawesi and Kalimantan), as well as Philippines and Cambodia. It is found in lowland forests and open areas (Uetz *et al*. 2016).

**Local remarks:** This species was observed sleeping, perched on the tips of tree branches, and camouflaged among dense foliage. It was easily located near human settlements and walkways leading to the Ms, as well as agricultural lands near Kts.

*Draco volans* (Linnaeus 1758) (Fig. 5E)

**Common name:** Common Flying Dragon

**Type locality:** N/A

**Distribution and habitat:** This species is found only on the Indonesian islands of Java and Bali. This agamid is found in submontane and lowland forest, as well as urban areas with an elevation of up to 1,500 m asl (Quah *et al*. 2018; Uetz *et al*. 2016).

**Local remarks:** On the tree, the flying lizard was observed. This species has been discovered in Mss.

#### Family Gekkonidae

*Cyrtodactylus marmoratus* (Gray 1831b) (Fig. 5F)

**Common name:** Marbled Bow-fingered Gecko

**Type locality:** Indonesia (Java)

**Distribution and habitat:** This species is found only on the Indonesian island of Java. They are found in lowland forest habitats (Mecke *et al*. 2016; Uetz *et al*. 2016).

**Local remarks:** The gecko was found on a mossy cliff in Kts, Kls and Ms.

*Gehyra mutilata* (Wiegmann 1834) (Fig. 5G).

**Common name**: Common Four-clawed Gecko, Stump-toed Gecko, and Stump-tailed Gecko

**Type locality:** Philippines (Manila)

**Distribution and Habitat:** The gecko is found throughout Indonesia (Sumatra, Kalimantan, Java, Bali and Nusa Tenggara), Malaysia, Philippines, New Guinea, Singapore, Thailand, Myanmar, Lao PDR, Vietnam and Cambodia, and was probably introduced to Mexico, Cuba, Hawaii, Mauritius, Seychelles and Madagascar. This species is strongly associated with primary forest habitats as well as human settlements (Uetz *et al*. 2016).

**Local remarks:** It is easily found around the exterior of Mv and Mss buildings.

*Gekko gecko* (Linnaeus 1758) (Fig. 5H)

**Common name:** Tokay Gecko, Tokeh-tokeh, and Tuctoo

**Type locality:** Indonesia (Java)

**Distribution and habitat:** This species is found throughout India, Bangladesh, Nepal, Bhutan, Thailand, Myanmar, Lao PDR, Cambodia, southern China, Vietnam, Malaysia, Philippines and Indonesia (specifically, Sumatra, Kalimantan, Java, Bali, Lombok, Sulawesi and Nusa Tenggara). Habitat is closely associated to the urban environment (Uetz *et al*. 2016).

**Local remarks:** The gecko was discovered hiding on the roof canopy surrounding settlements in Mv and on the trunks of large trees in Ms.

*Hemidactylus frenatus* (Duméril & Bibron 1836) (Fig. 6A)

**Common name:** Common House Gecko, Asian House Gecko, South Asian House Gecko, Spiny-tailed House Gecko, and Bridled House Gecko

**Type locality:** Indonesia (Java)

**Distribution and habitat:** The species is found throughout the tropics and subtropics. Its origins can be traced all the way back to Southeast Asia and the Indo-Australian archipelago. They are found in urban and forested areas and can be found up to 1,600 m asl (Uetz *et al*. 2016).

**Local remarks:** In Mss and Mv, the house gecko was common near human settlements.

#### Family Lacertidae

*Takydromus sexlineatus* (Daudin 1802) (Fig. 6B)

**Common name:** Asian Grass Lizard, Long-tailed Lizard, Six-striped Lizard, and Grass Lizard

**Type locality:** N/A

**Distribution and habitat:** It is distributed throughout South and Southeast Asia, including India, Thailand, China, Lao PDR, Myanmar, Vietnam, Cambodia, Malaysia and Indonesia (i.e., Sumatra, Java and Kalimantan). Habitats are associated with open areas, such as marshes and grassland, which are found primarily on mid-hills to low land up to an elevation of 850 m asl (Uetz *et al*. 2016).

**Local remarks:** This lizard was observed sleeping on top of the tall grass near the Mv’s walkway at night.

#### Family Scincidae

*Dasia olivacea* (Gray 1839) (Fig. 6C)

**Common name:** Olive Dasia, and Olive Tree Skink

**Type locality:** Malaysia (Penang Island)

**Distribution and habitat:** This species is found throughout South and Southeast Asia, including India, Myanmar, Thailand, Vietnam, Cambodia, Lao PDR, Malaysia, Philippines and Singapore (i.e., Sumatra, Kalimantan, Java and Bali). It is found in open forest at elevations of up to 1,200 m asl. It is commonly found on large trees, particularly in the buffer zone (Uetz *et al*. 2016).

**Local remarks:** This lizard was observed climbing the trunk of a tree near Mss, approximately 8 m above the ground.

*Eutropis multifasciata* (Kuhl 1820) (Fig. 6D)

**Common name:** Common Sun Skink, Many-lined Sun Skink, Common Mabuya, Javan Sun Skink and East Indian Brown Mabuya

**Type locality:** Indonesia (Java)

**Distribution and habitat:** This species is found throughout Indonesia (including Sumatra, Kalimantan, Java, Bali, Nusa Tenggara, Sulawesi and Papua), Bangladesh, India, China, Taiwan, Thailand, Cambodia, Lao PDR, Myanmar, Vietnam, Peninsular Malaysia, Singapore, Timor-Leste and New Guinea. This species is found in a wide variety of habitats, including peat swamp forest, montane forest, disturbed riparian habitats, moist lowland, tropical dry, agricultural land, savannah, eucalyptus forest, coffee plantations, woodland and gardens up to an elevation of 1,800 m asl (Uetz *et al*. 2016).

**Local remarks:** In Kts, Ds, and Ms, the skink was observed sleeping beneath leaf litter and tree trunks at night. It was also observed during the day.

*Eutropis rugifera* (Stoliczka 1870) (Fig. 6E)

**Common name:** Rough-scaled Sun Skink

**Type locality:** India (Nicobar Islands)

**Distribution and habitat:** It is distributed in India, Malaysia, Singapore, Philippines and Indonesia (i.e., Sumatra, Kalimantan, Java, and Bali). Habitats are associated with forested areas in the mid-hills (Uetz *et al*. 2016).

**Local remarks:** The skink was discovered in daylight above the fallen trees in Ms, and was also discovered hiding beneath leaf litter near the Ds.

#### Family Varanidae

*Varanus salvator* (Laurenti 1768) (Fig. 6F)

**Common name:** Common Water Monitor

**Type locality:** Indonesia (Java)

**Distribution and habitat:** This monitor lizard is found in Sri Lanka, India, Bangladesh, China, Thailand, Myanmar, Cambodia, Lao PDR, Vietnam, Malaysia, Singapore and Indonesia (i.e., Sumatra, Kalimantan, Java, Bali, Nusa Tenggara and Sulawesi), where it is mostly found in swamps and riverbanks (Uetz *et al*. 2016).

**Local remarks:** In the morning, a juvenile lizard was observed beneath a palm tree in Mv. In Kts, a sub-adult lizard was observed sleeping on tree branches. An adult was spotted swimming near a mangrove not far from Mss.

#### Family Colubridae

*Ahaetulla prasina* (Boie 1827) (Fig. 6G)

**Common name:** Asian Vine Snake, Oriental Whip Snake, Jade Vine Snake and Gunther’s Whip Snake

**Type locality:** Indonesia (Java)

**Distribution and habitat:** This species is found throughout Southeast Asia, including China, Philippines, India, Bangladesh and Sri Lanka. This snake is found in primary moist lowland and montane forests, secondary forests, open and dry forests, disturbed forests, scrublands, and plantations, as well as city gardens and urban areas (Uetz *et al*. 2016).

**Local remarks:** The snake was discovered on the tips of the branches, as well as in the shrubs surrounding the walkway to Ms.

*Boiga dendrophila* (Boie 1827) (Fig. 6H)

**Common name:** Yellow-ringed Cat Snake, Gold-ringed Cat Snake and Mangrove Snake

**Type locality:** Indonesia (Java)

**Distribution and habitat:** This species was widely distributed across Southeast Asia. It is found in Sumatra, Java, Kalimantan and Sulawesi of Indonesia. Their habitat is primarily lowland forests, specifically mangrove swamps and peat swamp forests, which reach elevations of up to 700 m asl (Uetz *et al*. 2016).

**Local remarks:** In Mss, an adult was observed slithering along drainage canals, heading towards a tree.

*Boiga nigriceps* (Günther 1863) (Fig. 7A)

**Common name:** Black-headed Cat Snake

**Type locality:** N/A

**Distribution and habitat:** It is found in Sumatra, Kalimantan, and Java of Indonesia. Additionally, it also occurs in China, Malaysia, and Thailand. Their natural habitats are primarily lowland forest (e.g., peat swamps) (Uetz *et al*. 2016).

**Local remarks:** This species was discovered in shrubs adjacent to dried streams in the Kts production forest.

*Dendrelaphis pictus* (Gmelin 1789) (Fig. 7B)

**Common name:** Common Bronze-back Snake, Painted Bronze-back Snake and Indonesian Bronze-back Snake

**Type locality:** *“Indiae orientalis”* = Southeast Asia

**Distribution and habitat:** It is found throughout Sumatra, Java and Kalimantan. This species inhabits a diverse range of habitats, including freshwater habitats such as slow-moving waters, rice fields, marshes, canals, and brackish water (Uetz *et al*. 2016).

**Local remarks:** The snake was found on a tree branch in Kts and Ms.

*Dendrelaphis underwoodi* (van Rooijen & Vogel 2008) (Fig. 7C)

**Common name:** Underwood’s Bronzeback Tree Snake

**Type locality:** Indonesia (West Java)

**Distribution and habitat:** This species is found only in West Java (i.e., Simpai mountain, Rajamandala, and Gulayang Province). This species is found in lowland to mid-hill forests at elevations ranging from 35 m asl–990 m asl (Uetz *et al*. 2016).

**Local remarks:** The snake was discovered near the tip of shrubs that act as canopies for the vegetation below, approximately 2 meters above the ground in Ms. This record extends the range of this species known to exist to Central Java.

*Lycodon subcinctus* (Boie 1827) (Fig. 7D)

**Common name:** Malayan Banded Wolf Snake

**Type locality:** Indonesia (Java)

**Distribution and habitat:** This species is found throughout Indonesia, Timor-Leste, Brunei Darussalam, Malaysia, Thailand, Lao PDR, Vietnam, Cambodia and China. This species is typically found in lowland forests and at elevations of up to 1,000 m asl (Uetz *et al*. 2016).

**Local remarks:** The snake was spotted slithering through the rocky stream near the Ms’s karst caves.

*Ptyas korros* (Schlegel 1837b) (Fig. 7E)

**Common name:** Chinese Ratsnake and Indo-Chinese Rat Snake

**Type locality:** Indonesia (Java)

**Distribution and habitat:** This species occurs throughout Indonesia, Singapore, Malaysia, Thailand, Myanmar, Cambodia, Lao PDR, Vietnam, China, Taiwan, Bangladesh, India and Bhutan. The snake can be found in lowland to montane forests at elevations up to 3,000 m asl (Uetz *et al*. 2016).

**Local remarks:** This species was discovered on the outskirts of estuaries, on the tips of mangroves. It was observed on the Mss’s northern flank.

#### Family Elapidae

*Bungarus candidus* (Linnaeus 1758) (Fig. 7F)

**Common name:** Malayan Krait and Blue Krait

**Type locality:** N/A

**Distribution and habitat:** This venomous species is found in Southeast Asia, specifically in Indonesia, Malaysia, Thailand, Vietnam, Cambodia and Lao PDR. It is found in lowland to submontane forests. It is also found in urban and agricultural areas up to 1,525 m asl (Uetz *et al*. 2016).

**Local remarks:** A juvenile individual was discovered near the stream near the Mv to Mss walkway. In Kls, Kts, and Ms, an adult was also observed slithering around the stream’s edge.

#### Family Homalopsidae

*Enhydris enhydris* (Schneider 1799) (Fig. 7G)

**Common name:** Rainbow Water Snake, Rainbow Mud Snake, Smooth Water Snake and Striped Water-Snake

**Type locality:** “*Indiae orientalis*” = Southeast Asia

**Distribution and habitat:** It is widely distributed in Indonesia, Singapore, Malaysia, Thailand, Vietnam, Cambodia, Myanmar, India, Nepal and Bangladesh. It is found predominantly in freshwater habitats, such as slow-moving streams, rice fields, canals and brackish water (Uetz *et al*. 2016).

**Local remarks:** This water snake was encountered in a muddy field in Mss that had previously been used as a fishpond.

#### Family Pareidae

*Pareas carinatus* (Wagler 1830) (Fig. 7H)

**Common name:** Keeled Slug-Eating Snake

**Type locality:** Indonesia (Java)

**Distribution and habitat:** This species is found throughout Asia and Southeast Asia, particularly in China, Myanmar, Thailand, Cambodia, Vietnam, Lao PDR, Malaysia and Indonesia (i.e., Sumatra, Java, Kalimantan, Bali and Nusa Tenggara). This species is found in lowland to submontane forests at elevations ranging from 550 m asl–1,300 m asl (Uetz *et al*. 2016).

**Local remarks:** This small snake was spotted in the shrubs near the stream. Their presence was established by the abundance of their prey (i.e., slugs) in the Ms.

#### Family Pythonidae

*Malayopython reticulatus* (Schneider 1801) (Fig. 8A)

**Common name:** Reticulated Python

**Type locality:** Malaysia (Rengit)

**Distribution and habitat:** This large constrictor snake is found throughout Southeast Asia, including Indonesia, Philippines, Malaysia, Thailand, Lao PDR, Vietnam, Myanmar, Cambodia and Bangladesh. It is found in a variety of habitats, including primary and secondary forests, savannah, shrublands, wetlands, peat swamps, mangrove swamps, grasslands, agricultural areas and urban areas. This species is found at elevations ranging from 0 m asl–1,300 m asl (Uetz *et al*. 2016).

**Local remarks:** In Mv, an adult was discovered hiding in the bushes not far from a human settlement.

#### Family Typhlopidae

*Indotyphlops braminus* (Daudin 1803) (Fig. 8B)

**Common name:** Brahminy blindsnake, Bootlace Snake and Flowerpot Snake

**Type locality:** India

**Distribution and habitat:** Although this blind snake is believed to have originated in Asia or Africa, it has been introduced to many continents, including Europe, America, Oceania, and Australia. It is frequently encountered in both populated and forested areas up to an elevation of 0 m asl–2,000 m asl (Uetz *et al*. 2016).

**Local remarks:** This blind snake was found in Mv on walkways near a human settlement.

#### Family Xenodermidae

*Xenodermus javanicus* (Reinhardt 1836) (Fig. 8C)

**Common name:** Dragon snake, Xenodermine Snake and Java Tubercle Snake

**Type locality:** Indonesia (Java)

**Distribution and habitat:** It has been reported to occur in Indonesia as well as other Southeast Asian countries such as Myanmar, Thailand, and Malaysia. They inhabit lowland forests and agricultural areas between 500 m asl and 1,100 m asl (Uetz *et al*. 2016).

**Local remarks:** Above the mossy rocks, an adult individual was discovered. In Ms, the snake had recently finished eating *Leptobrachium hasseltii*. Additionally, we discovered juveniles above the leaf litter near the Ds.

### Community, Diversity and General Observations

#### Distribution across sites and vegetational types

**Figure 9:**
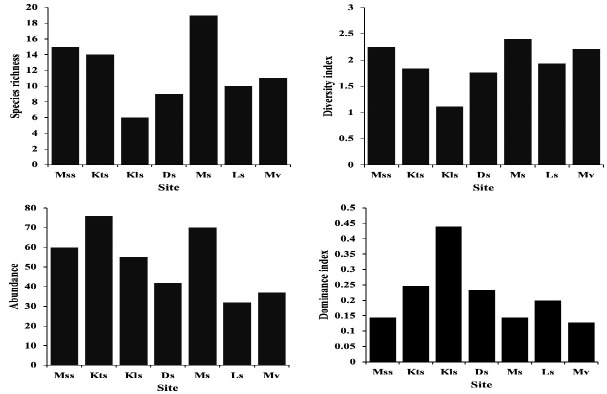
Species richness, diversity index, abundance, and dominance index of herpetofauna community in the surveyed sites on the western part of Nusa Kambangan Island. Abbreviations: (Mss) Masigit Sela stream, (Kts) Ketapang stream, (Kls) Kalidua stream, (Ds) Darmoko stream, (Ms) Mangunjaya stream, (Ls) Lapas stream, (Mv) Motean village.

A total of 372 individuals comprising of 43 herpetofauna species were collected from the western part of Nusa Kambangan Island, from various sites, habitat complexes and/or vegetational types. Fig. 9 shows diversity indices consisting of species richness, diversity index, abundance, and dominance index based on the surveyed sites. Ms had the highest number of species richness (19), whereas Kls had the lowest number of species richness (6). Most sites show moderate diversity index (H’: 1–3). However, Ms had the highest diversity index (2.399), whereas Kls had the lowest diversity index (1.113). Zar’s modified *t*-tests or Hutcheson *t*-tests show that some of diversity index differs significantly between sites (Fig. 9; Table 2). Kts and Ms had the highest abundance (Fig. 9) due to the high number of *P. leucomystax* (Kts: 28 individuals, Ms: 13 individuals) and *C. chalconota* (Kts: 24 individuals, Ms: 21 individuals). Kls had the highest dominance index (0.44) due to high number of two species aforementioned (≥ 20 individuals), however, the dominance in most of the surveyed sites can be considered as low (D: ≤ 0.50).

**Figure 10:**
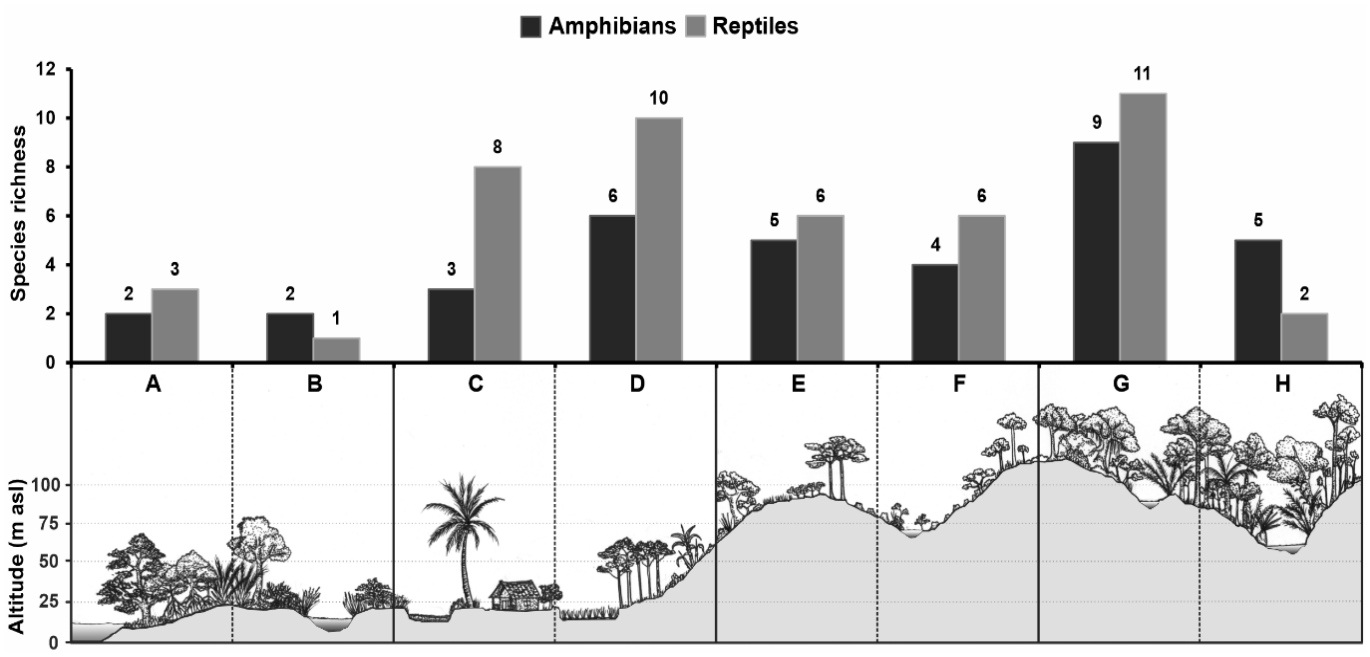
Species richness of amphibians and reptiles distributed in the western part of Nusa Kambangan Island. Habitat complexes and/or vegetation types as follows: (A) mangrove forest type 1, (B) mangrove forest type 2, (C) fishpond and settlement area, (D) paddy field and timber production forest, (E) edges between agriculture and forest, (F) tributary stream and degraded areas, (G) lowland forest type 1, and (H) lowland forest type 2 (see Materials and Methods for details).

Lowland forest type 1 had the highest species richness with 20 species (11 reptiles, 9 amphibians) associated with the presence of specialists such as *R. reinwardtii, C. dentata, A. cartilaginea*, and *X. javanicus*, followed by paddy field and timber production forest with 16 species (10 reptiles, 6 amphibians) but mostly consisting of generalists such as *F. cancrivora, F. limnocharis, C. chalconota, P. leucomystax, B. jubata, E. multifasciata, A. prasina*, and *D. pictus* (Fig. 10; Table 3). The mangrove forest type 2 had the lowest species richness, with only 3 species (1 reptiles, 2 amphibians). Species richness varied across habitat complexes and/or vegetation types.

The average air temperature is between 26°C–30°C, and the average relative humidity is between 85%–100% (Table 4). Mss was the most humid site due to recent rain, but also the driest due to the unavailability of water bodies. The remainders depict varying degrees of mean air temperature and mean humidity, with Ms yielding the most species. The majority of herpetofauna species discovered during our survey were predominantly associated with streams, rivers, and areas adjacent to water bodies.

## DISCUSSION

According to our surveys, the total herpetofauna species comprised 21% of the herpetofauna species found in Java (i.e., 47 amphibians by Frost 2020; 161 reptiles by Uetz *et al*. 2016). Comparing species richness directly across Java sites is impractical due to the size difference in the surveyed area, the disparity in sampling efforts, and the differences in sampling skills and experiences (Kurniawan *et al*. 2021; Kusrini *et al*. 2021). However, comprehending the herpetofauna’s diversity generally requires such direct comparisons. Kadafi *et al*. (2020) discovered a total of 38 species of herpetofauna in Kondang Merak forest, one of the few remaining lowland rainforests in Java. Their surveys listed 8 amphibians, 15 lizards, and 15 snakes. Milto and Lukin (2020) examined species diversity in Ujung Kulon National Park, one of the few remaining areas in Java with a primary lowland rainforest. They found 21 amphibian and 65 reptile species, 15 of which (17%) were newly discovered, updating the previous list. These demonstrates that the amphibian and reptile fauna of Java’s lowland forests is diverse and waiting to be found.

*Kalophrynus minusculus*, a rare microhylid previously restricted to West Java’s Ujung Kulon National Park (Zug 2015), has been confirmed in our studies, which require detailed examination because detailed morphology, morphometry, and molecular data have never been reported (Iskandar 1998; Zug 2015). Our surveys confirmed the presence of *Dendrelaphis underwoodi*, which was previously thought to be restricted to West Java (Uetz *et al*. 2016). This demonstrates that numerous herpetofauna species are likely waiting to be discovered, and with additional surveys, particularly in the eastern part of Nusa Kambangan Island, we expect discovering additional herpetofauna species not previously reported.

Java retains only a few pristine areas as a result of rapid population growth, which exacerbates forest degradation and land conversion (Kurniawan *et al*. 2021). This will eventually leave small patches of natural habitat capable of supporting lowland forest herpetofauna (Namkhan *et al*. 2020), such as Nusa Kambangan Island. This island is home to a sparse population of residents who are spread throughout the region. The majority of residents rely on the island’s natural resources, which, if depleted, could be detrimental not only to the herpetofauna, but also to the environment, overall biodiversity, and residents. Unfortunately, this island is impacted by human activities such as limestone mining (Ayuningrum & Purnaweni 2018), illegal logging and fuel wood chopping (Robiansyah & Davy 2015), and mangrove habitat degradation (Hariyadi & Madduppa 2018).

Another way to aid conservation efforts on Nusa Kambangan Island could be to conduct research on ecologically significant species (Mills *et al*. 1993). Given the inevitable anthropogenic threats, community-based conservation may be a much preferable option for ensuring biodiversity’s welfare and sustainability (Berkes 2007). For instance, residents of Nusa Kambangan Island rely heavily on natural resources such as clean water obtained from streams. Additionally, protecting water sources may also protects habitats for highly sensitive species such as *C. dentata* and *A. cartilaginea*. Disseminating information about their ecosystem services may alter residents’ perceptions and help them avoid overexploitation of natural resources. Numerous herpetofauna species, including *P. leucomystax* (tolerance for disturbed habitat) and *R. reinwardtii* (usually found in pristine areas), can be used as bioindicators of environmental change or habitat disturbance. Additional research is required to ensure the effectiveness of community-based conservation, including local attitudes, participation, conservation awareness, and good governance.

## CONCLUSION

Our initial surveys on Nusa Kambangan Island’s west part resulted in the identification of 43 herpetofauna species (16 amphibians and 27 reptiles). Our analyses indicate that Nusa Kambangan Island is capable of supporting suitable habitats for a diverse herpetofauna, but the habitats have been threatened by human disturbance. Community-based conservation efforts may provide an alternative method of conserving the island’s biodiversity. We hope to encounter additional herpetofauna species in the future through additional surveys, particularly in the eastern part of Nusa Kambangan Island.

## Figures and Tables

**Table 1:** List of herpetofauna species found on the western part of Nusa Kambangan Island.

Taxa	Survey site							Conservation Status		

	Mss	Kts	Kls	Ds	Ms	Ls	Mv	IUCN	CITES	PERMEN LHK
Bufonidae										
*Duttaphrynus melanostictus*	–	–	–	–	–	–	**√**	LC	•	•
*Ingerophrynus biporcatus*	√	–	–	–	–	–	–	LC	•	•
*Leptophryne borbonica*	–	–	√	–	–	–	–	LC	•	•
Dicroglossidae										
*Fejervarya cancrivora*	√	–	–	–	–	–	√	LC	•	•
*Fejervarya limnocharis*	√	√	–	√	–	–	√	LC	•	•
*Limnonectes kuhlii*	–	√	–	–	√	–	–	LC	•	•
*Limnonectes macrodon*	–	√	–	–	√	–	–	LC	•	•
*Limnonectes microdiscus*	–	–	–	√	√	–	–	LC	•	•
*Occidozyga lima*	–	√	–	–	–	√	–	LC	•	•
Megophrydae										
*Leptobrachium haseltii*	–	–	–	–	√	√	–	LC	•	•
Microhylidae										
*Kalophrynus minusculus*	–	–	√	–	–	√	–	LC	•	•
*Microhyla achatina*	√	–	–	–	–	√	–	LC	•	•
Ranidae										
*Indosylvirana nicobariensis*	–	–	–	√	–	√	–	LC	•	•
*Chalcorana chalconota*	√	√	√	√	√	√	–	LC	•	•
Rhacophoridae										
*Polypedates leucomystax*	√	√	√	√	√	√	–	LC	•	•
*Rhacophorus reinwardtii*	–	–	–	–	–	√	–	NT	•	•
Geoemydidae										
*Cyclemys dentata*	–	√	–	√	√	√	–	NT	II	•
Tryonicidae										
*Amyda cartilaginea*	–	–	–	–	–	√	–	VU	II	•
Crocodilidae										
*Crocodylus porosus*	√	–	–	–	–	–	–	LC	II	P
Agamidae										
*Bronchocela jubata*	–	√	–	–	√	–	–	LC	•	•
*Draco volans*	√	–	–	–	–	–	–	LC	•	•
Gekkonidae										
*Cyrtodactylus marmoratus*	–	√	√	–	√	–	–	LC	•	•
*Gehyra mutilata*	–	–	–	–	–	–	√	NE	•	•
*Gekko gecko*	–	–	–	–	√	–	√	LC	II	•
*Hemidactylus frenatus*	√	–	–	–	–	–	√	LC	•	•
Lacertidae										
*Takydromus sexlineatus*	–	–	–	–	–	–	√	LC	•	•
Scincidae										
*Dasia olivacea*	√	–	–	–	–	–	–	LC	•	•
*Eutropis multifasciata*	–	√	–	√	√	–	–	LC	•	•
*Eutropis rugifera*	–	–	–	√	√	–	–	LC	•	•
Varanidae										
*Varanus salvator*	√	√	–	–	–	–	√	LC	II	•
Colubridae										
*Ahaetulla prasina*	–	–	–	–	√	–	–	LC	•	•
*Boiga nigriceps*	–	√	–	–	–	–	–	LC	•	•
*Boiga dendrophila*	–	–	–	–	–	–	–	NE	•	•
*Dendrelaphis pictus*	–	√	–	–	√	–	–	NE	•	•
*Dendrelaphis underwoodi*	–	–	–	–	√	–	–	LC	•	•
*Lycodon subcintus*	–	–	–	–	√	–	–	LC	•	•
*Ptyas korros*	√	–	–	–	–	–	–	NE	•	•
Elapidae										
*Bungarus candidus*	–	√	√	–	√	–	√	LC	•	•
Homalopsidae										
*Enhydris enhydris*	√	–	–	–	–	–	–	LC	•	•
Pareidae										
*Pareas carinatus*	–	–	–	–	√	–	–	LC	•	•
Pythonidae										
*Malayopython reticulatus*	–	–	–	–	–	–	√	LC	II	•
Typhlopidae										
*Indotyphlops braminus*	–	–	–	–	–	–	√	NE	•	•
Xenodermidae										
*Xenodermus javanicus*	–	–	–	√	√	–	–	LC	•	•

*Notes*: Table entries include taxon family and species, annotated checklist of herpetofauna for each surveyed site ([Mss] Masigit Sela stream, [Kts] Ketapang stream, [Kls] Kalidua stream, [Ds] Darmoko stream, [Ms] Mangunjaya stream, [Ls] Lapas stream, [Mv] Motean village; presence = √, absence = –), and conservation status of herpetofauna according to IUCN (LC = Least Concern, VU = Vulnerable, NT = Near Threatened, NE = Not Evaluated), CITES (II = Appendix II, • = Not Evaluated), and PERMEN LHK (P = Protected, • = Not Protected).

**Table 2:** Summary of results of Zar’s modified *t*-tests/Hutcheson *t*-tests, comparing herpetofauna species diversity between surveyed sites on Nusa Kambangan Island, Central Java, Indonesia.

Site		H_x_	H_y_	t_cal._	t_crit._	Statistical value
Mss vs	Kts*	2.253	1.837	2.242	1.977	df = 136, *p* = 0.026
	Kls*	2.253	1.113	6.288	1.981	df = 114, *p* = 0.001
	Ds*	2.253	1.761	2.584	1.986	df = 92, *p* = 0.011
	Ms^ns^	2.253	2.399	0.799	1.978	df = 130, *p* = 0.425
	Ls^ns^	2.253	1.927	1.579	1.996	df = 67, *p* = 0.118
	Mv^ns^	2.253	2.212	0.245	1.985	df = 95, *p* = 0.806
Kts vs	Kls*	1.837	1.113	3.786	1.978	df = 129, *p* = 0.001
	Ds^ns^	1.837	1.761	0.379	1.982	df = 105, *p* = 0.704
	Ms*	1.837	2.399	2.918	1.976	df = 146, *p* = 0.004
	Ls^ns^	1.837	1.927	0.419	1.991	df = 77, *p* = 0.676
	Mv*	1.837	2.212	2.093	1.981	df = 110, *p* = 0.038
Kls vs	Ds*	1.113	1.761	3.309	1.986	df = 92, *p* = 0.001
	Ms*	1.113	2.399	6.822	1.979	df = 124, *p* = 0.001
	Ls*	1.113	1.927	3.850	1.994	df = 70, *p* = 0.001
	Mv*	1.113	2.212	6.294	1.986	df = 92, *p* = 0.001
Ds vs	Ms*	1.761	2.399	3.235	1.983	df = 101, *p* = 0.001
	Ls^ns^	1.761	1.927	0.757	1.995	df = 69, *p* = 0.451
	Mv*	1.761	2.212	2.450	1.991	df = 77, *p* = 0.016
Ms vs	Ls*	2.399	1.927	2.219	1.992	df = 74, *p* = 0.029
	Mv^ns^	2.399	2.212	1.064	1.983	df = 105, *p* = 0.289
Ls vs	Mv^ns^	1.927	2.212	1.419	2.001	df = 58, *p* = 0.160

*Notes*: (Mss) Masigit Sela stream, (Kts) Ketapang stream, (Kls) Kalidua stream, (Ds) Darmoko stream, (Ms) Mangunjaya stream, (Ls) Lapas stream, (Mv) Motean village.

* Significant difference between sites; p ≤ 0.05;

ns No significant difference between sites; p ≥ 0.05

**Table 3:** List of herpetofauna species found on the western part of Nusa Kambangan Island.

Taxa	Vegetation type and/or habitat complex							

	A	B	C	D	E	F	G	H
Bufonidae								
*Duttaphrynus melanostictus*	*–*	*–*	*√*	*√*	*–*	*–*	*–*	*–*
*Ingerophrynus biporcatus*	*–*	*–*	*–*	*–*	*√*	*–*	*–*	*–*
*Leptophryne borbonica*	*–*	*–*	*–*	*–*	*–*	*–*	*–*	*√*
Dicroglossidae								
*Fejervarya cancrivora*	√	√	√	√	–	–	–	–
*Fejervarya limnocharis*	√	√	√	√	√	√	–	–
*Limnonectes kuhlii*	–	–	–	–	–	–	√	–
*Limnonectes macrodon*	–	–	–	–	–	√	√	–
*Limnonectes microdiscus*	–	–	–	–	–	–	√	–
*Occidozyga lima*	–	–	–	–	–	–	√	–
Megophrydae								
*Leptobrachium haseltii*	–	–	–	–	–	–	√	√
Microhylidae								
*Kalophrynus minusculus*	–	–	–	–	–	–	–	√
*Microhyla achatina*	–	–	–	√	√	–	–	–
Ranidae								
*Indosylvirana nicobariensis*	–	–	–	–	–	–	√	–
*Chalcorana chalconota*	–	–	–	√	√	√	√	√
Rhacophoridae								
*Polypedates leucomystax*	–	–	–	√	√	√	√	√
*Rhacophorus reinwardtii*	–	–	–	–	–	–	√	–
Geomydidae								
*Cyclemys dentata*	–	–	–	–	–	–	√	√
*Tryocnicidae*								
*Amyda cartilaginea*	–	–	–	–	–	–	√	–
*Crocodilidae*								
*Crocodylus porosus*	√	–	–	–	–	–	–	–
Agamidae								
*Bronchocela jubata*	–	–	√	√	√	√	–	–
*Draco volans*	–	–	–	√	–	–	–	–
Gekkonidae								
*Cyrtodactylus marmoratus*	–	–	–	–	–	√	√	–
*Gehyra mutilata*	–	–	√	√	–	–	–	–
*Gekko gecko*	–	–	√	–	–	–	–	–
*Hemidactylus frenatus*	–	–	√	√	–	–	–	–
Lacertidae								
*Takydromus sexlineatus*	–	–	–	√	–	–	–	–
Scincidae								
*Dasia olivacea*	–	–	–	–	–	–	√	–
*Eutropis multifasciata*	–	–	–	√	√	√	√	–
*Eutropis rugifera*	–	–	–	–	–	–	√	–
Varanidae								
*Varanus salvator*	√	–	√	–	–	√	–	–
Colubridae								
*Ahaetulla prasina*	–	–	–	√	√	–	–	–
*Boiga dendrophila*	–	√	–	–	–	–	–	–
*Boiga nigriceps*	–	–	–	–	–	√	–	–
*Dendrelaphis pictus*	–	–	–	√	√	√	√	–
*Dendrelaphis underwoodi*	–	–	–	–	–	–	√	–
*Lycodon subcinctus*	–	–	–	–	–	–	√	–
*Ptyas korros*	√	–	–	–	–	–	–	–
Elapidae								
*Bungarus candidus*	–	–	√	–	√	–	√	√
Homalopsidae								
*Enhydris enhydris*	–	–	√	–	–	–	–	–
Pareidae								
*Pareas carinatus*	–	–	–	√	√	–	–	–
Pythonidae								
*Malayopython reticulatus*	–	–	–	√	–	–	–	–
Typhlophidae								
*Indotyphlops braminus*	–	–	√	–	–	–	–	–
Xenodermidae								
*Xenodermus javanicus*	–	–	–	–	–	–	√	–

*Notes*: Table entries include taxon family and species, annotated checklist of herpetofauna for each habitat complex and/or vegetation type as follows: (A) mangrove forest type 1, (B) mangrove forest type 2, (C) fishpond and settlement area, (D) paddy field and timber production forest, (E) edges between agriculture and forest, (F) tributary stream and degraded areas, (G) lowland forest type 1, and (H) lowland forest type 2; presence = √, absence = – (see Materials and Methods for details).

**Table 4:** Mean air temperature and humidity for each surveyed site in the western part of Nusa Kambangan Island.

Survey site	Mean Air Temperature (± °C)	Mean Humidity (± %)
Masigit Sela stream (Mss)	30	100
Ketapang stream (Kts)	27	85
Kalidua stream (Kls)	26	98
Darmojo stream (Ds)	29	92
Mangunjaya stream (Ms)	28	90
Lapas stream (Ls)	27	90
Motean village (Mv)	28	95

## APPENDIX

List of specimens deposited in Laboratory of Ecology and Animal Diversity, Department of Biology, Universitas Brawijaya (NK)

*Ingerophrynus biporcatus:* NK 0117

*Leptophryne borbonica:* NK 1983, NK 1984, NK 1985, NK 1986, NK 1987

*Fejervarya cancrivora:* NK 0118

*Fejervarya limnocharis:* NK 0116

*Limnonectes kuhlii:* NK 1949

*Limnonectes macrodon:* NK 1948

*Limnonectes microdiscus:* NK 1951

*Occidozyga lima:* NK 0106

*Leptobrachium hasseltii:* NK 1854, NK 1855, NK 1856, NK 1857

*Kalophrynus minusculus:* NK 1978, NK 1979

*Microhyla achatina:* NK 1956, NK 1957, NK 1958, NK 1959, NK 1960, NK 1961, NK 1962, NK 1963, NK 1964, NK 1965, NK 1966, NK 1967

*Indosylvirana nicobariensis:* NK 1964

*Chalcorana chalconota:* NK 1858

*Polypedates leucomystax:* NK 1963

*Rhacophorus reinwardtii:* NK 1870, NK 1871

*Cyclemys dentata:* NK 0160, NK 0161, NK 0162, NK 0163

*Bronchocela jubata:* NK 0111, NK 0112

*Cyrtodactylus marmoratus:* NK 1958

*Gekko gecko:* NK 0109, NK 0110

*Hemidactylus frenatus:* NK 0125, NK 0126

*Takydromus sexlineatus*: NK 01730, NK 0174

*Eutropis multifasciata:* NK 1959, NK 1960

*Ahaetulla prasina:* NK 0092, NK 0093, NK 0094, NK 0095

*Boiga dendrophila:* NK 1980

*Dendrelaphis pictus:* NK 1981

*Dendrelaphis underwoodi:* NK 1863

*Lycodon subcintus:* NK 1982

*Bungarus candidus:* NK 1841

*Enhydris enhydris:* NK 0123, NK 0124

*Pareas carinatus:* NK 1842

*Indotyphlops braminus* NK 2060

*Xenodermus javanicus:* NK 1860, NK 1861
